# An interstitial deletion at 8q23.1-q24.12 associated with Langer-Giedion syndrome/ Trichorhinophalangeal syndrome (TRPS) type II and Cornelia de Lange syndrome 4

**DOI:** 10.1186/s13039-015-0169-9

**Published:** 2015-08-12

**Authors:** Nikoletta Selenti, Maria Tzetis, Maria Braoudaki, Krinio Gianikou, Sofia Kitsiou-Tzeli, Helen Fryssira

**Affiliations:** Department of Medical Genetics, Aghia Sophia Childrens’ Hospital, Athens University, School of Medicine, Thivon and Levadeias 11527, Goudi, Athens, Greece

## Abstract

**Background:**

There are three distinct subtypes of Trichorhinophalangeal syndrome (TRPS); TRPS type I, TRPS type II and TRPS type III. Features common to all three subtypes include sparse, slowly growing scalp hair, laterally sparse eyebrows, a bulbous tip of the nose (pear-shaped), and protruding ears. Langer–Giedion syndrome (LGS) or TRPS type II is a contiguous gene syndrome on 8q24.1, involving loss of functional copies of the *TRPS1* and *EXT1* genes. We report a male patient that was referred to the Department of Medical Genetics due to hypotonia and dysmorphic facial features.

**Results:**

Cytogenetic and array- Comparative Genomic Hybridization (aCGH) analysis revealed that the patient was a carrier of an interstitial deletion at 8q23.1-q24.12 of 12,5 Mb. Parental karyotype indicated that the father carried an apparently balanced insertion: 46, ΧΥ, der(10)ins(10;8)(q22;q23q24).

**Conclusions:**

This is the first report of an apparently balanced insertion including chromosomes 8 and 10 contributing to the etiology of LGS/ TRPS type II. Τimely diagnosis of parental balanced chromosomal rearrangements can reduce the risk of subsequent miscarriages as well as abnormal offspring.

## Background

Langer–Giedion syndrome (LGS) or Trichorhinophalangeal syndrome (TRPS) type II is a contiguous gene syndrome on 8q24.1, involving loss of functional copies of the *TRPS1* and *EXT1* genes, that was first described by Andreas Giedion, a Swiss pediatric radiologist. In 1966, he reported the association of slowly growing hair, a long pear-shaped nose with a bulbous tip, and finger deformities. In 1969, Giedion A. and Leonard O. Langer Jr. independently described a patient with these features as well as multiple exostoses. In 1974, the name LGS or TRP II was introduced subsequently by Hall et al. [1]. The diagnosis of TRPS type I, II and III is based on clinical and radiographic features as well as on genetic analysis that is helpful especially in the case of non-classical clinical presentation.

- TRPS type I caused by mutations in the *TRPS1* gene located on chromosome 8q24.1, characterized by distinctive skeletal abnormalities and craniofacial dysmorphism.
- TRPS type II represents a contiguous gene syndrome involving loss of functional copies of the *TRPS1* and *EXT1* genes and is characterized by multiple exostoses and intellectual disability (ID) in addition to the classical triad of symptoms.
- TRPS type III is caused by mutations in *TRPS1* gene and includes severe short stature and brachydactyly in the absence of exostoses [2].

It is postulated that nonsense mutations are usually responsible for the TRPS type I phenotype, while missense mutations cause the TRPS type III phenotype [3]. The mode of inheritance in TRPS I and III is autosomal dominant. In contrast, TRPS II (LGS) is usually sporadic and caused by the deletion of contiguous genes in the long arm of chromosome 8 (8q24.11-13), involving loss of functional copies of the *TRPS1* and the *EXT1* genes [4, 5].

In TRPS I and III, the eyebrows may be thickened medially and thin or even absent laterally. In TRPS II, the eyebrows could be normal. Sparse eyelashes and secondary sexual hairs may be evident. A long pear-shaped nose with bulbous tip and a long philtrum are characteristic. Deformities of the fingers and toes can result from cone-shaped epiphyses and irregular premature fusion, which could be mistaken as juvenile rheumatoid arthritis [1]. The middle phalanges are most commonly involved. Sometimes there is an abnormal patella with recurrent dislocation. Perthes-like change of the hip is common and can lead to severe secondary osteoarthritis in the adult [1, 6]. In TRPS II, patients have additional features of multiple exostoses from early childhood, microcephaly, skin and joint laxity. Mental impairment is common.

Features common to all three disorders include sparse, slowly growing scalp hair, laterally sparse eyebrows, a bulbous tip of the nose (pear-shaped), and protruding ears. Further typical characteristics comprise a long flat philtrum and a thin upper vermillion border. Distinct radiographic findings are often not detectable before 2 years of age.

## Clinical Phenotype/Genotype correlation in LGS/TRPS II

The LGS/TRPII comprises the clinical features of two separate autosomal dominant diseases : the multiple hereditary exostoses (MHE) type I caused by mutation in the gene encoding exostosin-1 (EXT1), and the TRPS type I caused by haploinsufficiency of the *TRPS1* gene. The MHE is a genetically heterogeneous disorder which can be caused by mutations in the *EXT1*, *EXT2* or *EXT3* gene [7].

The LGS/TRPSII shows some clinical variability depending on the loss of additional genes in the deleted region [8, 9]. Mild to moderate intellectual disability, congenital nephrotic syndrome [10], hydrometrocolpos [11], conductive hearing loss [12], growth hormone deficiency [13], persistent cloaca and prune belly sequence [14], and a submucous cleft palate [15] have all been described as additional features of LGS/TRPSII depending on the deletion size.

Although deletions of the 8q region are rare and considerably variable, the shortest region of deletion overlap (SRO) has been defined at 8q24.1, spanning 2 Mb, including *TRPS1*, *EIF3S3*, *RAD21, OPG, CXIV* and *EXT1* genes [16].

Heterozygous mutations of the *RAD21* gene at 8q24.11 were recently found to cause Cornelia de Lange syndrome- 4 (CdLs-4), which is characterized by growth deficiency, mental retardation, microcephaly, bushy eyebrows and synophrys, depressed nasal bridge, micrognathia, micromelia, hearing loss, anteverted nares, prominent symphysis and spurs in the anterior angle of mandible, gastrointestinal problems (such us gastroesophageal reflux, duplication of the gut, malrotation of colon with volvulus and pyloric stenosis). There are occasional seizures, congenital heart defects and inguinal hernias [17–19].

TRPS type I, LGS/TRPSII and CdLs-4 share many facial features; some patients affected by LGS/TRPSII with deletions involving *RAD21* and not *TRPS1*, present typical facial dysmorphisms. LGS/TRPSII patients’ facial hallmarks might be partially influenced by *RAD21*. Moreover, the phenotypic consequences of *RAD21* disruption might be underestimated in these patients [20].

## Parental apparently-balanced chromosomal insertion in the etiology of LGS/TRPSII

Structural aberrations in human chromosomes can be initiated by chromosomal breakage and incomplete repair. If the chromosomal aberration is balanced, copy number for all interrogated genomic regions is disomic. However, most chromosomal aberrations are unbalanced, resulting in duplication or deletion of genetic material at the chromosomal breakpoints. The frequency of insertional translocation is estimated by karyotyping on severely mentally retarded patients (4:40,000) [21]. Microscopically visible insertional translocations occur with a frequency of 1:80.000 in newborns [22]. However, the frequency is also estimated as high as 1:500 using array-based comparative genome hybridization (aCGH) as a detection method in conjunction with FISH [23].

The frequency of balanced chromosomal insertions is much lower than reciprocal translocations because it requires three chromosomal breaks. Parental balanced insertions increase the possibility of copy number imbalances in the offspring resulting in congenital anomalies of multiple organ systems and psychomotor retardation. aCGH analysis revealed that approximately 2.1 % of parents with offspring having developmental anomalies, harbor balanced insertions [24]. In this regard, genomic analysis of the parental DNA of a patient may help us to exclude the possibility of parental balanced translocations as predisposing genetic risk.

We report an LGS/TRPSII patient with an 8q23.1-q24.12 microdeletion, caused by a parental apparently balanced insertion ( 46,XY, der(10)ins(10;8)(q22;q23q24).

## Case presentation

### Clinical report

Our male patient was the first child of healthy unrelated parents of normal intelligence. He was born by caesarian section at 40 weeks of gestation after an uneventful pregnancy. His birth weight was 3090 gr (25^th^ centile), length 49 cm (25^th^ centile) and head circumference 33 cm (25^th^ centile). Previously the mother had suffered a miscarriage.

**Fig. 1:**
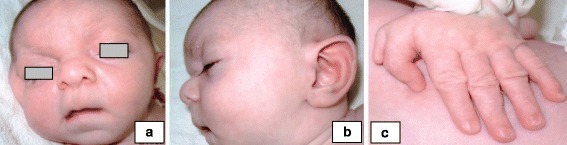
Dysmorphic features in a LGS patient, that was referred to our Department of Medical Genetics. **a** A bulbous tip of the nose, a long and flat philtrum and a thin upper lip vermilion, **b** large laterally protruding ears and a depressed and broad nasal bridge and **c** pre-axial polydactyly

He was referred to our Department of Clinical Genetics at 2 months due to hypotonia and dysmorphic facial features. On clinical examination the patient presented with brachycephaly, low anterior hairline, a depressed and broad nasal bridge, a bulbous tip of the nose, a long and flat philtrum, a thin upper lip vermilion, a high palate, retro-micrognathia and downslanting palpebral fissures (Fig. 1a). He had large laterally protruding ears (Fig. 1b), bilateral pre-axial polydactyly (Fig. 1c), long fingers and cryptorchidism. Heart and abdominal ultrasound revealed no pathological abnormalities. The brain MRI showed dysplastic corpus callosum. The opthalmological examination was normal. The karyotype was found to be abnormal [46, XY, del(8)(q23 → q24) pat].

## Methods

### Cytogenetic analysis

Cytogenetic analysis was performed on GTG-banded metaphases at a resolution of 500 bands approximately. The samples were subjected to blood lymphocyte culture according to the standard cytogenetic protocol. The description of karyotypes were made in accordance with the ISCN 2013 (International System of Human Cytogenetic Nomenclature).

### Array-CGH analysis (aCGH)

After informed consent from the parents, genomic DNA from the patient and the father was obtained from 3 ml of peripheral blood using the BioRobot® M48 System (Qiagen, Hilden, Germany) and the commercially available kit MagAttract® DNA Blood Midi M48 Kit (Qiagen). The quality and quantity of the DNA samples was determined using a NanoDrop ND-1000 UV–VIS spectrophotometer. Agilent Human Genome 4X180K CGH + SNP arrays were used in this study (Agilent Technologies, Santa Clara, CA, www.agilent.com). Labelling and hybridization was carried out according to the manufacturer’s recommendations. Data were processed using Feature Extraction and analysed using Cytogenomics vs.3.0 software (Agilent Technologies, CA) with the following settings: Algorithm: ADM-1, Threshold: 6.7, with a minimum of 4 probes for a region to be included. Centralization and fuzzy zero corrections were applied to remove putative variant intervals with small average log_2_ ratios.

## Results

### Cytogenetic analysis

**Fig. 2:**
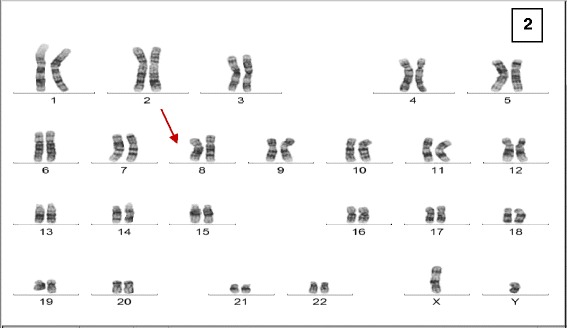
Cytogenetic analysis. G-banded chromosomal analysis from the peripheral blood of the LGS patient displayed a male karyotype with an apparent deletion on one chromosome 8q (46,XY, del(8)(q23 → q24)pat)

G-banded chromosomal analysis from the peripheral blood of the patient displayed a male karyotype with an apparent deletion on one chromosome 8q (46,XY, del(8)(q23 → q24)pat) (Fig. 2). The maternal karyotype was normal (46,XX), however the paternal karyotype revealed an apparently balanced insertion: 46, ΧΥ, der(10)ins(10;8)(q22;q23q24).

### Array-CGH analysis (aCGH)

**Fig. 3:**
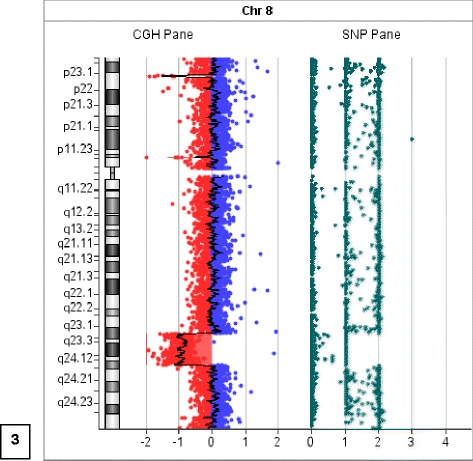
Molecular cytogenetic analysis. Whole genome array-CGH revealed an interstitial deletion at 8q23.1-q24.12 of 12,5 Mb (HGVS:NC_000008.10:g.(?_108,776,506)_(121,312,414_?)del; GRCh37/hg19) in our LGS patient

Whole genome a-CGH revealed an interstitial deletion at 8q23.1-q24.12 of 12,5 Mb (HGVS:NC_000008.10:g.(?_108,776,506)_(121,312,414_?)del; GRCh37/hg19). The deletion included the following genes: *RSPO2, EIF3E, TTC35, TMEM74, TRHR, NUDCD1, ENY2, PKHD1L1, EBAG9, SYBU, KCNV1, CSMD3, MIR2053, TRPS1, EIF3H, UTP23, RAD21, NCRNA00255, MIR3610, C8orf85, SLC30A8, MED30, EXT1, SAMD12, TNFRSF11B, COLEC10, MAL2, NOV, ENPP2, TAF2, DSCC1, DEPTOR, COL14A1* (Fig. 3)*.* The paternal result included only copy number polymorphisms (CNPs), therefore the father carried a balanced insertion.

## Discussion and conclusions

Parental balanced chromosomal rearrangement is an important cause of copy number alteration in the offspring. A recent study revealed that approximately 2.1 % of duplications and deletions associated with developmental delay originated from parental balanced chromosomal aberrations [24]. Repeat sequences, such as low copy repeats (LCR) around the region of breakage are thought to mediate misalignment between the repeat regions during meiosis, leading to unequal recombination events [25]. However, the incidence of cytogenetically-visible insertions is low [21], and the potential parental carrier status cannot be easily detected. Parental FISH analysis as well as aCGH should be considered as part of the clinical baseline testing, in all families, where a de novo interstitial aberration is suspected. By identifying parental interstitial aberrations, both prenatal and preimplantation genetic counseling should be offered to reduce the risk of subsequent miscarriages as well as chromosomally abnormal offspring [26].

We were able to demonstrate that using a combination of karyotyping and aCGH, an insertion involving chromosomes 8 and 10 in a phenotypically normal father caused LGS/TRPSII in the proband. The 8q23.1-q24.12 deletion of 12.5 Mb, contains 33 genes of which *TRPS1, EXT1,* and *RAD21* could be considered responsible for the core phenotypic features of the proband. Although deletions of the 8q region are rare and considerably variable, the shortest region of deletion overlap (SRO) has been identified as spanning 2 Mb, including *TRPS1, EIF3S3, RAD21, OPG, CXIV, EXT1* genes [16]. To our knowledge, this is the first report of an apparently balanced insertion including chromosomes 8 and 10 contributing to the etiology of LGS/TRPSII.

Recently, defects in *RAD21* have been associated with CdLs-4. Although recognizable, the face and hair of patients with LGS/TRPSII are variable and typically related to *TRPS1* disruption [27]. In our patient, aCGH analysis revealed the deletion of both *TRPS1 and RAD21* genes which is in accordance with the dysmorphic facial features. TRPS type I, LGS/TRPSII and CdLs-4 share many facial features. Autosomal dominant missense mutations result in more severe functional, structural and cognitive clinical findings compared to loss-of-function mutations or deletions. In *RAD21* haploinsufficiency or heterozygous loss-of-function mutation may cause the LGS/TRPSII affected individuals to have mild CDLS stigmata too [19].

In LGS/TRPSII patients, the more distal the proximal breakpoint is located from the *TRPS1* gene, the fewer typical features of LGS/TRPSII are observed [28]. In that case, the *TRPS1* gene expression may be altered if the proximal breakpoint occurs near the gene where a potential regulatory sequence, such as gene enhancer or repressor is located. Thus, the location of the proximal breakpoint causes a functional disturbance of the *TRPS1* gene. The size of the critical region outside the *TRPS1* gene which contains the regulatory sequence is yet to be determined. Another possibility is that the newly formed chromosomal imbalance may cause a silencing effect on the *TRPS1* gene by placing it in close proximity to another gene [29].

Nearly 70 % of LGS/TRPSII affected individuals may exhibit mild to severe cognitive disability [30]. Although delay in the LGS/TRPSII syndrome has been attributed to the deletion of genes outside the *TRPS1-EXT1* interval, specific genes have yet to be identified. The size of the 8q deletion is directly correlated with intellectual disability (ID)[13].

In our patient, the 8q deletion spans 12,5 Mb and the genes potentially involved were: *CSMD3, MED30, SAMD12, EIF3H, RAD21* and *TAF 2*. In particular, *CSMD3* has previously been suggested as a good candidate for pathogenesis of developmental delay in LGS/TRPSII patients. *RAD21* mutations or deletions have been observed in CdLs patients with very mild intellectual disability (ID) [19]. Mutations in the *TAF2* gene are also related with ID [31, 32].

Although more than 100 patients with LGS/TRPSII have been reported in the literature, there is little long-term follow-up and knowledge about the course and potential further health complications during adulthood and advanced age. In our patient, clinical reevaluation at six months revealed feeding difficulties and delayed psychomotor development.

The number of distinct syndromes due to specific chromosome imbalance is rapidly increasing, especially since a-CGH allows detection of submicroscopic aberrations of virtually any segment. Confirmation of LGS/TRPSII using the appropriate genetic analysis is necessary especially in cases of non-classical clinical presentation [33].

Skeletal and orthopedic problems continue to be an important issue. Severe scoliosis and orthopedic problems due to joint laxity, Perthes disease, asymmetry of legs and joint immobility due to adjacent exostoses are among the most frequent problems. On the other hand, eye, ear, and cardiac problems are rarely of major relevance [33].

Τimely diagnosis of parental balanced chromosomal rearrangements using the appropriate techniques is important and can reduce the risk of subsequent miscarriages as well as abnormal offspring; in our case neither parental karyotype nor amniocentesis were performed for prenatal testing. The monosomy 8q23-q24 observed in the affected child resulted from segregation during paternal meiosis, due to the apparently balanced chromosomal insertion in the father 46, ΧΥ, der(10)ins(10;8)(q22;q23q24).

With better knowledge on the long-term course, complications and molecular defects that cause LGS/TRPSII, genetic counseling is necessary. Clinical geneticists should provide information for the families, to advise them about the course of the disease and how to overcome problems [33].

## Consent

Written informed consent was obtained from the patient for publication of this Case report and any accompanying images. A copy of the written consent is available for review by the Editor-in-Chief of this journal.
