# Erratum to: An interstitial deletion at 8q23.1-q24.12 associated with Langer-Giedion syndrome/ Trichorhinophalangeal syndrome (TRPS) type II and Cornelia de Lange syndrome 4

**DOI:** 10.1186/s13039-015-0174-z

**Published:** 2015-09-30

**Authors:** Nikoletta Selenti, Maria Tzetis, Maria Braoudaki, Krinio Giannikou, Sofia Kitsiou-Tzeli, Helen Fryssira

**Affiliations:** Department of Medical Genetics, Aghia Sophia Childrens’ Hospital, Athens University, School of Medicine, Thivon and Levadeias, 11527 Goudi Athens, Greece

The original version of this article [1] unfortunately contained a mistake. In the author list, the surname of author Krinio Giannikou was incorrectly spelled. The original article has now been updated with the correct spelling.
